# Military Surgery

**Published:** 1821-06-01

**Authors:** 


XIII.

I. *A Treatise on Gun-Shot Wounds, on Injuries of Nerves, and on Wounds of the Extremities requiring the different Operations of Amputation; in which the various Methods of performing these Operations are shown, together with their After-treatment; and containing an Account of the Author's successful Case of Amputation at the Hip-Joint, &c. &c. &c. With five explanatory Plates. Being a Record of the Opinions and Practice of the Surgical Department of the British Army, at the Termination of the War in Spain, Portugal, and France, in* 1814. *The Second Edition, considerably enlarged.* By G. J. Guthrie, Deputy inspector of Hospitals during the Peninsular War; Surgeon to the Royal Westminster Infirmary for Diseases of the Eye; Consulting Surgeon to the Western Dispensary for the Diseases of Women and Children; Member of the Medical and Chirurgical Society of London; Associate of the Medical Societies of the Faculty of Paris, and of Aberdeen; Lecturer on Surgery, &c. One vol. octavo, pp. 526. London, 1820.

II. *Principles of Military Surgery, &c. &c.* By John Hennen, M.D. F.R.S.E. Deputy Inspector of Hospitals. *Second Edition.* Edinburgh, 1820.

III. *Dictionaire des Sciences Medicales, Tom.* 43, *Art.* "*Plaies D'Armes à feu.*" Par M. M. Laurent et Percy.

---

——————— factorum est copia nobis,
Res gestæ Regumque Ducumque et Tristia Bella.

"Je vais dans les loisirs d'une paix, helas! trop certaine, preparer, si je puis, de nouveaux secours aux guerriers." *Percy.*

---

The revolutionary war has furnished ample materials to swell the medical as well as the military annals of Europe. In this long and sanguinary conflict, man was but too often placed in situations which were calculated to call forth the whole energies of his nature; and obstacles, previously considered insurmountable, were overcome with a celerity—it might be almost said, with a *facility*, truly astonishing. In our days, armies from the North were seen marching in the hottest season of the year, over the burning sands of Suez —while those raised on the luxurious shores of the Mediterranean were braving a Russian winter on the snow-covered plains of Moscow. Whole fleets of LINE-OF-BATTLE SHIPS have rushed forth from the ports of Britain, in the very depth of winter, and fearlessly steered their course amid the dangerous syrtes of the Rhine and the Scheldt, regardless of the tremendous storms which assailed them from the West, or the still more formidable fields of ice which came drifting on them from the East. In this eventful period the mental and corporeal powers of British soldiers and sailors were put to the test of ultimate exertion in every clime. They were exposed to all the "skiey influences" of the torrid and the frozen zone, in addition to the havoc of warfare, and the ravages of disease. Did the medical officers of our fleets and armies participate in the moral and physical excitement produced by these portentous scenes around them? Unquestionably they did. That grand parent of invention— NECESSITY, was ever at work, prompting measures to obviate rising difficulties, by which means these officers ascertained the real extent of the powers of Nature and Art in a far more accurate manner than ever could be done in private life, where, not only a multiplicity of means are used, but a multiplicity of counteracting agents are perpetually interfering and leading to error. No one is now so unjust as to deny the numerous improvements which medicine and surgery have derived from the observations and experience of practitioners in the public service of their country; and nothing can be more clear than that the professional as well as the general public are daily appreciating the value of that hard-earned knowledge reaped by the meritorious class of officers in question, and now applied to the exigencies of private life.

Mr. Guthrie has long been well known as a medical officer who has profited by the extensive field of experience which the campaigns of the immortal Wellington presented to his view, amid the mountains of the Peninsula, and on the plains of Waterloo; and he has communicated the result, not only of his own personal experience, which must have been very extensive; but also "that of the whole of the officers during several (peninsular) campaigns." In this way Dr. Hennen and himself have laid the surgical profession under the deepest obligations, for the assiduity with which they observed themselves, and the industry with which they collected the observations of others, on all the important points of military, and indeed we might add, civil surgery. The works of these two officers form a lasting trophy to the *surgery* of the late war, and much do we hope that military *medicine* may be enriched by some similar publications, in the same manner as a Desgenettes and a Broussais have acted in France. The practitioners of the Army, Navy, and Company's service have greatly extended our knowledge of the diseases affecting our countrymen between the tropics and in the warm latitudes of the Mediterranean; but the diseases from which our troops must have often suffered in the Peninsular war, are yet without a historian, excepting Dr. Somers on Dysentery, and some detached papers in periodical publications.

Mr. Guthrie, with a very laudable attention to the convenience of his brethren, has published the *entirely new* part of the present work separately, for the accommodation of those who purchased his work on "wounds of the extremities requiring amputation," published a few years ago. It is proper to observe, however, that in reprinting the *latter* work, he has made some important additions, which we shall notice in the proper place. Mean time our analytical labours must be directed almost entirely to the *first* or new part of the volume before us, which is occupied with the important subject of gun-shot wounds in general and in particular.

When arrows were exchanged for ball-cartridge the lodgment of these latter projectiles in the human body caused great alarm in the minds of our chirurgical brethren of the fourteenth century; and the idea of such wounds being poisoned, led to dressings (boiling oil for instance) more tormenting than the original injury. An accidental want of oil discovered to Ambrose Paré the cruelty of this mode of treatment, but it was long before a rational one was substituted in its stead. To this day some erroneous notions respecting gun-shot wounds continue to prevail. For instance, being *contused* wounds they have been supposed to be comparatively free from pain and hæmorrhage, and to be almost inevitably attended with suppuration or sloughing—errors, Mr. Guthrie observes, which operate with fatal influence on the practice growing out of them. The *degree* of contusion depends on the shape of the projectile, the force of its impulsion, and the resistance opposed, the symptoms and appearances being influenced accordingly. Neither are gun-shot wounds painless, at the moment of infliction, as some have supposed. In some instances the pain is trifling, being, cæteris paribus, in an inverse ratio to the degree of contusion. In fact, however, the sensation of pain varies almost with every shade of idiosyncrasy of constitution:—"for in two persons suffering apparently from the same kind of injury, and with the same detriment, one will writhe with agony, whilst the other will smile with contempt." Generally speaking, the greater the velocity with which the projectile is impelled—the rounder and smaller the size—and the less the resistance opposed, so much the less will be the sensation of pain produced by the accident.

The subject of hæmorrhage is one of much greater importance than that of pain. There is generally some bleeding attending gun-shot wounds, even where no vessel of calibre is divided. In wounds of the face and neck the hæmorrhage is generally considerable. If the ball happen to strike any hard substance, so as to be flattened or angulated, before entering the human body, the wound will be lacerated rather than contused, and the loss of blood probably the greater.

"The colour of the blood will be arterial, but a wound of a large artery is not to be feared, unless it be emitted in great quantity, and per saltum, and continues to be poured out of a bright colour and without intermission, in spite of the common pressure, or means made use of for its suppression; in which case the lesion of an artery of some magnitude is indicated. The bleeding from a simple flesh wound soon ceases, and does not return, except some violence be done to the part; whilst, in a case of wounded artery, it sometimes continues until the patient dies, which is frequently the case when a large artery is partially divided." P. 6.

If the artery be completely divided, a considerable quantity of blood is quickly lost, and syncope generally arrests the flow, though death may be the consequence in some cases. This syncope, for the most part, takes place when a limb is carried away, and dangerous hæmorrhage usually ceases before there is time for a tourniquet—nor does it generally return:—if it do return, the patient's life will be lost, unless assistance be on the spot. It will not, Mr. G. repeats, usually return, after spontaneous cessation, unless from impropriety of conduct or accidental violence—"neither will a due degree of suppuration or sloughing in the wound effect it."

"The chance of secondary hæmorrhage is but trifling; and if in such cases as these it is so, there can be no doubt but that it must be next to nothing in others where no vessel of importance has been injured. On the separation of the sloughs, a little blood may occasionally be lost, but it is generally caused by the impatience of the surgeon, or the irregularity of the patient, and seldom requires attention. Sometimes at this period, that is, from the eighth to the twentieth day, a large artery will give way from sloughing or ulceration; but the proportion of cases requiring the ligature of arteries, will not be greater than three or four in a thousand taken indiscriminately, exclusive of hæmorrhage caused by hospital gangrene, inordinate sloughing, or broken bones, which are not the usual causes hitherto alluded to as inducing secondary hæmorrhage; and which, as they may almost always be avoided by proper care and management, cannot with propriety be considered, as legitimate causes." 9.

Even where the elasticity of the artery enables it to recede (when not hit direct) with the injury of some of its coats, it does not necessarily follow that sloughing and hæmorrhage shall be the result. Mr. G. has known instances where the coats inflame, and the canal of the vessel is obliterated. He has a preparation where a ball passed between the femoral artery and vein, without dividing either; and, in other instances, he has reason to believe, the vessel completely recovers itself. In fine, our experienced author concludes that the opinion, *that gun-shot wounds do not bleed at the moment of infliction, unless a large artery be cut—and that they generally bleed, and often profusely, after suppuration*, cannot be too soon banished from the minds of surgeons, as leading to unnecessary anxiety and bad practice.

Our surgical readers are aware, that much discussion took place a few years ago, respecting the propriety of *immediate* amputation in cases of gun-shot wounds requiring that operation, and that as an argument for this measure, it was asserted that no constitutional shock, as it was termed, took place in cases of wounds from cannon shot—consequently there was no necessity for a moment's delay. Mr. G. is of a different opinion.

" When an organ of importance has been injured, and the blow severe, as by a cannon or grape shot, or shell, or from the fracture of a bone, and even from the attention being directed to the receipt of an injury from the situation in which the soldier may be placed, a peculiar constitutional alarm ensues in a much greater degree than would follow an injury of equal magnitude, precisely in the same spot, from any other cause. It affects alike, although not in an equal manner, the coward and the brave, the man of learning and the unlettered soldier. As it is, however, an affection often greatly augmented from an association of ideas, so is it more immediately controlled by men of sound judgment, or command of mind, and is more certainly removed by the knowledge of the injury being of little consequence." 10.

To this subject we shall return a little farther on. In the mean time, as Mr. Guthrie properly observes, the continuance of the constitutional alarm or shock ought to excite great suspicion of serious injury, and render us guarded in our prognosis. Here Mr. Guthrie relates several interesting cases, happening in various ranks, illustrative of this commotion, and proving that the infliction of a gun-shot wound of any importance, is *generally* followed by more or less hæmorrhage, pain, a peculiar anxiety, alarm, and loss of animal and organic powers, characteristic of this kind of injury.

As a ball very commonly carries in with it some portion of clothes or other extraneous substance, it is desirable to ascertain if it have passed out again—and yet it is not always possible to decide on which is the exit or which the entrance of the missile. The position of the man, at the time of receiving the wound, will much facilitate this judgment. The most marked peculiarity attending the entrance of the ball is "a circular depression capable of admitting the little finger, and of a livid colour; whilst the exit is more ragged, not depressed, sometimes little more than a slit or rent." These appearances, however, are by no means constant, or so strongly marked—especially if the velocity, and, consequently, impetus of the ball be great; for then the entrance and exit will often be considerably similar. But we must pass over a great deal of curious and interesting matter, relative to the course, lodgment, and ejection of balls, detailed from page 20 to 27, and to which the surgical reader ought to pay the greatest attention. We come now to the mode of treatment; and here Mr. Guthrie wisely directs the surgeon to a consideration of the patient's constitution, as well as the local injury, for inattention to this point, he thinks, has caused much of the discrepancy of practice in gun-shot wounds.

The ball, in its rapid course through a muscular part, deprives of sensibility, or even life, the immediate track of the missile. These contused portions, and the blood extravasated among them, must be thrown off by the surrounding healthy parts, leaving, of course, a track much larger than at the moment of injury. Inflammation is the process of Nature, set up for the repair of the injured parts, and on the due management of this, our good or bad success depends. The difference, however, in constitutions must always require a great latitude in the treatment. People between 15 and 40 years of age can, in general, best bear the inflammation and fever attending wounds, and the treatment necessary to regulate them. In good constitutions the increased action may be so balanced by moderate bleeding, that fever and inflammation may go on for several days, and terminate without detriment.

"In bad constitutions this is impossible, for if evacuations are at all indicated, they must be effected in the first days, I might even say, hours, for the patient will otherwise be unable to bear them; his powers will sink, and the fever, or inflammation, will be brought to an unfavourable termination. A good constitution can bear a high action and a direct reduction of power, for a sufficiently long time. A bad constitution can bear neither for half the same period; and if the reduction of the action is to be attempted by reducing the power, (that is, by bleeding), it must be done at the commencement. Persons of bad habits will almost always bear bleeding once. Old people bear it much better. I am quite certain, from long experience, that many of both descriptions are continually lost, from a want of decision in practice, in the first instance. An ulterior state of disease is too much regarded, and the consequence is, that we have so many unfortunate sequelæ." 37.

In our *systematic* works sixteen ounces of blood were, till lately, generally ordered to be abstracted in acute inflammatory diseases of the brain, heart, lungs, stomach, intestines, &c. But, as Mr. Guthrie justly observes, this quantity will not suffice. In good constitutions it may mitigate, but its repetition even will not remove the disease. Army and navy practitioners have, of late years, altered the opinions of the profession, and demonstrated a bolder and a safer track. So far back as 1801, Mr. Guthrie was led to adopt decisive depletion in acute diseases, from the bad success attending the then common treatment, as laid down in books, and the ravages of disease apparent on dissection. In a digression on peritonæal inflammation, Mr. Guthrie seems to question the propriety of considering diseases as differing according to the different structures of which the organs are composed—" without due attention to their intimate connexion and mutual dependence on each other." No one can doubt that disease readily passes from one structure to another; but the fact of the structures modifying the nature of the disease, is now put beyond all manner of doubt. What practitioner has not seen disease confined for weeks and months to the mucous membrane of the bowels, without any affection whatever of the peritonæal covering, and *vice versa?* In our article on peritonæal inflammation (No. 2.) we stated it thus:—

" Nothing is more certain than that almost every acute inflammation of the organs above-mentioned, begins in a single tissue of their structure, with corresponding symptoms, and spreads thence to other tissues, with more or less rapidity, according to the violence of the disease, and the mode of treatment, accompanied also by a train of phenomena indicative of the new structures successively invaded." P. 162.

We do not see any reason to alter this statement, which is the one Mr. Guthrie has objected to; but it is necessary to observe, that by *peritonæal* inflammation we never meant inflammation of that portion of the membrane reflected along the inner surface of the abdominal parietes, but that portion, and that portion solely, which is reflected over the stomach, intestines, &c. forming, to all intents and purposes, as regular a constituent part of their structure as the subjacent muscular or mucous coats. This being explained, we believe Mr. Guthrie and ourselves are precisely of similar sentiments, both in the pathology and treatment of this dangerous class of diseases. We are particularly happy, too, in having the corroboration of Mr. Guthrie's opinion respecting a point which we contested with Dr. Abercrombie. " Acute inflammation," says our author, " of the peritonæum covering the bowels does never take place without the bowels being costive." Mr. Guthrie asks here, what is the diagnostic symptom which indicates this degree of enteritic inflammation from that which involves the other structures of the gut? We answer, that he himself has furnished the diagnosis—*constipation.* Did Mr. Guthrie ever see a case where the *mucous* structure of the intestine was inflamed, primarily or secondarily, without diarrhœal or dysenteric symptoms? We are bold to say he never did. Where, then, is the impropriety of investigating the separate affections of these membranes, seeing that they do really often exist in nature—though often indeed blended, especially in the progress of disease. Here Mr. Guthrie relates a very curious case of inflammation of the stomach ending in an effusion of a pus-like matter into the cellular texture of the organ, which, on dissection, was found so changed from its natural appearance, as to resemble the thick smooth part of boiled tripe. It must be observed, however, that this disease is so very rare, as not to be taken into consideration in actual practice. Speaking of diagnostic symptoms, we shall introduce the following passage corroborative of an observation made by Dr. Dickson in page 166 of our second number of this series.

"If there be a symptom more observable than another, it is one which is yet common to all vital organs. I mean anxiety. The able observations of Dr. Dickson, of Clifton, noticed by Dr. Johnson, allude to this, although they more properly refer to the appearance of the countenance, as indicative of pain. The anxiety I intend to distinguish as a symptom, I have alluded to in dangerous wounds, and is not only of the mind, as shewn by the countenance in a very expressive manner, but of the body, as demonstrated by great uneasiness. Anxiety is constantly observed towards the fatal termination of acute diseases; but when a vital organ is affected, whether it be its internal structure or not, this symptom is often present at an earlier period. In some cases it is a more certain sign, than the pulse, of great derangement; in others, more distinctive than pain, which is sometimes referred to a part that is unaffected, and is, in all, indicative of the greatest danger, and demonstrative of the necessity of corresponding exertion on the part of the practitioner. In the case of gastritis, which I have related, the pain was referred so steadfastly to the region of the navel, that enteritis was looked upon as the principal disease, whilst no sign of it could be discovered, after death, in the parts corresponding to it." 47.

Mr. Guthrie states, that in the year 1813, he met with several fatal cases of inflammation of the heart, in hard drinkers, some from metastasis of rheumatism. In none of these were the symptoms well marked. "Indeed," says our author, "inflammation of the heart frequently exists without being suspected, and more especially when combined with inflammation of the chest." We know, from some experience, that in no organ is inflammatory action more frequently or more completely masked than in the central organ of the circulation.

"There is one symptom I have remarked, however, in an extraordinary degree, in all the cases I have seen, and that is *extreme anxiety*; and in a much greater degree than other symptoms would appear to warrant, or than can be accounted for from the apparen nature of the disease. In obscure cases of inflammatory affection of the chest, I consider it more constant, more pathognomonic of carditis, than fainting or irregularity of the pulse." 48.

Similar observations have been made by gentlemen in this and other countries. We cannot indeed wonder, that great anxiety and great *mental despondency* (a symptom very generally accompanying disease of the heart) should be produced, when a viscus so essential to existence is labouring in its function. Here Mr. Guthrie relates a case shewing the necessity of our being on our guard when this anxiety is present, without adequate apparent cause.

" A soldier applied to me for an external injury, labouring at the same time under some internal complaint, for which he had been recommended to take bark, on the supposition that it was an ague. I found on inquiry that the paroxysms were not regular, that the rigors were only slight shiverings, the heat was permanent, the skin dry, the sweating stage absent, pulse from 112 to 120, and a peculiar expression of anxiety of countenance that could not be accounted for. From this symptom I conceived that bark was improper, that the disease was not ague, that something more serious was impending: my opinion was not received, the bark was continued, wine mixed with water was allowed, but the man did not amend. The rigors, it is true, were suspended, and so was the bark, but the skin remained dry and harsh, the pulse always quick, sometimes full, the anxiety constant, the body rather wasting, although the appetite was better than could be expected. In this state he was removed to the country, for the benefit of the air, the complaint being considered nervous, the abdomen indicating no signs of derangement on pressure, nor the chest on inspiration. I still predicted an explosion, which would develop the mischief, and gave a caution on the subject. This development occurred suddenly, in the shape of a paralytic affection of one side, and examination after death showed the formation of an abscess on the opposite hemisphere of the brain. He would have been reckoned a rash and ignorant man, who would have advised the abstraction of blood from this patient at an early period of the complaint. I believe, reasoning from the result, there are none of us, who would not have done it. A few ounces of blood from the jugular vein might have saved the life of a valuable man."* 50.

Here Mr. Guthrie very truly observes, that inflammation seldom

---

" * Bleeding from the jugular vein is, in my opinion, much more effectual in all cases of internal derangement of the head, than from the temporal artery, or any other part. I believe it to be particularly so in cases which may be denominated chronic, or in low obscure inflammations or congestions. I have several people attending at my Infirmary for Diseases of the Eye, in whom the advantages of it have been most striking. In some cases of fits resembling epilepsy, it has put a stop to the disease. In epilepsy itself it has been useful, and in amaurosis from pressure on the brain, in congestion, and in impending paralysis, it has been of the most marked utility, when other general and local bleedings have failed."

runs so high and so rapid a course in foreigners, especially the natives of the warmer climates, as in our own countrymen—consequently the necessity for depletion is never *so* great in them, and hence their inert practice does pretty well with the subjects of it. The following extract, though long, cannot be abridged, and we do not like to pass it by unnoticed, as we consider it worthy of attention.

"Suppose two persons, of a middle age, residents in a confined part of London, addicted to full living, and what I consider as one of the marks of a very bad habit, corpulent, and of a sallowish complexion, and tinted with red vessels on the cheeks. Suppose that these persons as nearly resemble each other as possible, and that both are attacked (to carry on the argument) by inflammation of the peritonæum. In one, the symptoms shall be of enteritis, a *constant* hot burning pain at the region of the navel, increased by paroxysms, but never absent, augmented and rendered unbearable by pressure, constant motion of the body, which is never at ease, nausea and vomiting, anxiety of countenance, bowels confined, pulse quick, perhaps hard or uncertain or variable. In the other, the symptoms shall be of peritonitis; the pain less urgent, the general uneasiness and anxiety more moderate, the derangement of stomach not extending, or seldom amounting to vomiting; the pulse quick, the bowels may be either confined or open. In these cases we have acute inflammation in two persons, whose constitutions are equal to no continuance of exertion, not to say an increase of it; because an increase for a short period many of them can bear without detriment. Is is not of advantage to name these complaints differently, that we may not for a moment lose sight of the leading feature of the disease, the seat of it? Have we not a great advantage in having it constantly before us, that there is a difference in the mode of treatment? It is true we treat diseases according to symptoms, not according to the name we bestow upon them; but if there be an argument in favour of distinguishing diseases from each other, that have a general similarity, I know of none more strong than that which rests on difference of symptoms, and a marked difference in practice. In the first case of well-marked enteritis, we will suppose that we have had it in our power to ascertain, from previous illness, that the patient bears bleeding very badly, that the abstraction of twenty-four ounces of blood is likely to bring on dropsy, or the greatest general debility, even terminating in death. In such case, what is to be the practice? are we to allow the disease or the physician to kill the patient, or to combine the efforts of both, by applying leeches and fomentations to the belly, giving purgatives and glysters, and then attributing his death to his badness of constitution? A person in this state is precisely in the situation of a man who has sustained a serious injury of an extremity, requiring amputation. It must be done at the moment, or it never will be done. He is capable for a short time of bearing a great increase of action, but he is totally incapable of supporting it for any length of time. A person of this description, attacked in a warm climate by fever, which is not quickly cut short, almost invariably dies. Whatever is to be done must be done quickly. The patient with enteritis must be bled to the greatest extent that he can bear with safety. I consider twenty ounces taken at once, to be more efficacious than thirty ounces drawn at three periods; the effect on the constitution will be more immediate, more productive of benefit, and less permanently injurious. In the first case, there will be only loss of blood to contend with, inducing debility; in the second, both loss of blood and the effects of protracted disease; which last is in such cases by far the greater evil, inasmuch as it is generally a fatal one, from the mischief done to some organ or organs of vital importance.

"The patient with peritonitis, although in danger, is yet less urgently so, than he who is labouring under enteritis. The quantity of blood drawn need not exceed ten ounces, and great reliance may be placed on purgatives, especially on calomel, with opium, and blisters to the abdomen—remedies which are alike of little use in well-marked enteritis, until the inflammatory symptoms have been relieved by blood-letting." 52.

When amputation is performed on habits of the above description, the great attack is on the nervous system, which is least capable of bearing it. The fever is first inflammatory, tending daily to typhus mitior, or nervosa, ultimately proving fatal, with every appearance, in many cases, of derangement of the biliary organs. At the first moment of injury, Mr. G. observes, the operation should be performed, so that the shock to the nervous system may be, if possible, continuous, and opium with purgatives should be administered to allay it. When the reaction rises permanently, then the surgeon requires resolution, and at the same time, discrimination, to keep it in check, without running into the opposite extreme of lowering too far the powers of life. By some of Mr. Hunter's adherents, cordials, stimulants, and opiates, are continued, instead of active purgatives and saline medicines. Blood-letting is generally admissible; it is urgently demanded, "when the pulse becomes quick, and in any degree full, and followed by an opiate it is frequently decisive." The quantity, in these cases, must be moderate—from twelve to sixteen ounces. In some cases blood-letting cannot be ventured on—in a very few instances cordials and opiates will be more proper throughout. Cold is here injurious as an external application—and if amputation be performed, the stump should be led to suppuration, if possible.

Mr. Guthrie is happy to corroborate the practice of immediate amputation (where the operation is necessary) by the opinions of Mr. Astley Cooper and Mr. James Wilson. We do not deem it necessary to enter into the question whether the citizens of London should be exempted from the rule of immediate amputation where necessary. We cannot imagine what claim they can have for such an exemption. That the sedentary citizen will not bear an operation so well as a healthy soldier or sailor, we can easily imagine; but we think it quite evident that neither will he so well bear the constitutional fever and irritation which must ensue, if secondary amputation be preferred.

In simple gun-shot wounds, happening in sound constitutions, there is a strong tendency to run rapidly through the adhesive inflammation, and terminate in suppuration, unless gangrene should take place. The suppuration is generally in proportion to the inflammation, and our great object is to keep the inflammatory action in due bounds, so as to moderate the suppuration, and thus render the wound more disposed to heal. The idea entertained by some that the whole track of a gun-shot wound must suppurate and throw off a slough of dead matter, is erroneous. Until the peninsular war poultices were commonly applied to facilitate the suppuration and separation, a principle of practice laid down by John Hunter. But in this celebrated war poultices were found inconvenient to the practitioner, and not beneficial to the patient—cold water was therefore adopted in their stead, " and in many cases, lint and sticking plaster without the assistance of either." After a fair investigation, then, and repeated trials, our author hesitates not to affirm that the cold water is the best application—the inflammation being, in some instances, materially prevented, in many, greatly controlled, and in almost all, very much subdued by it, whilst the suppurative process is not impeded in the generality of cases, in a degree sufficient to interrupt the subsequent one of granulation. In flesh wounds, then, a piece of lint dipped in oil or spread with ointment, is to be put on the part, and secured by two cross pieces of adhesive, over which a compress or some folds of linen are to be placed, and kept constantly wetted with cold water, or even ice, if agreeable to the feelings of the patient. A roller is of no use, except to prevent the compress from shifting its position during sleep. In other respects it is injurious, as preventing the parts from swelling, and thus causing irritation. Under this plan the first dressing is not to be removed for two, three, or four days. It should then be softened with warm water, or an evaporating poultice,* before removal. Mr. Guthrie has a most decided aversion to poultices in wounds of any kind. In spontaneous suppurations they are useful—but after injuries, injurious, as the object is not so much to promote suppuration as to restrain inflammation, and the succeeding suppuration. Cold water, he repeats, is the proper application to gun-shot wounds in healthy constitutions. It is not, however, infallible, or always advantageous. There are many persons with whom it will disagree—and still more with whom it will disagree, after a certain time, in which cases, cold should not be persevered in. It does no good, in any stage of inflammation, when it produces disagreeable sensations in the patient. This generally takes place about the period of suppuration, and then fomentations are proper, with poultices, if convenient.

" In the spring of the year, the marsh-mallow makes an excellent

---

* " An evaporating poultice is made by crumbling some stale bread into a basin, and pouring boiling water upon it. The basin is then to be turned over on a plate, and the water allowed to drain off, when the poultice is ready for use." 64.

poultice, and so do turnips, gourds, carrots, &c. independently of oatmeal, linseed meal, Indian meal, or other farinaceous substances. In all those cases where a poultice is resorted to, as much attention is to be paid to the period of removing, as of applying, it. It is used to alleviate pain, stiffness, swelling, the uneasiness arising from cold, and to encourage the commencing or interrupted action of the vessels towards the formation of matter; and as soon as the effect intended has been gained, the poultice should be abandoned, and recourse again had to cold water, with compress and bandage." 67.

These applications should not be too general, lest they cause the patient to take cold. The parts only which are injured, and their immediate vicinity, are to be wetted—the rest of the body being kept perfectly dry. It is seldom expedient to apply cold to the trunk of the body. If the wound be simple, it is unnecessary; if any viscus be affected or cavity opened, external applications are of very subordinate consideration. In injuries of the head, however, it is otherwise.

When the slough is thrown off, or the parts begin to granulate, a compress and bandage will complete the cure, generally within the first six weeks.

" A great number are always cured within the first four, and in these cases bleeding is never thought of. Purgatives are occasionally administered, and abstinence is an excellent remedy; but bleeding, purgatives, occasional emetics, and starvation, are remedies of great importance; if the patients be irregular in their personal habits, or the inflammatory symptoms run high." 68.

Here Mr. Guthrie gives a very correct delineation of the progress of a gun-shot wound in healthy muscular parts, from the first day of infliction to the final cicatrization—a process scarcely requiring the interference of art, and producing but little constitutional disturbance. This favourable proceeding, however, is not to be expected in every instance. The state of constitution, the distresses of military warfare, exposure to the inclemency of the weather, or imprudence on the part of the patient, will frequently induce a train of untoward symptoms in wounds apparently of the same nature.

" After the first three days, the symptoms gradually increase, the swelling is much augmented, the redness spreads far from the edges of the wound, the pain becomes severe and constant. The wound remains dry, stiff, painful, with glistening edges. The sensibility is now much increased. The system sympathizes, the skin becomes hot and dry, the tongue loaded, the head aches, the patient is restless and uneasy, the pulse full and quick, there is fever of the inflammatory kind. The swelling of the part increases from the deposition of lymph and serum in the cellular membrane to a considerable extent above and below the wound, which is in a state of high inflammation; and, instead of being entirely superficial or confined to the immediate track of the ball, is spreading wide and deep in the muscular parts of the limb. The wound itself can hardly bear to be touched, it discharges but little, and the sloughs separate slowly. If the inflammation be manageable, matter begins to be secreted copiously, not only in the track of the wound, but in the surrounding parts; sinuses form in the course of the muscles or under the fascia, considerable surgical treatment is necessary, and the cure is protracted from three to four and even six months, and is often attended for a longer period with lameness from contraction of the muscles or adhesions of the cellular membrane. The parts, from having been so long in a state of inflammation, are much weaker; and if the injury has been in the lower extremity, the leg and foot swell on any exertion, which cannot be performed without pain and inconvenience for a considerable time; the inflammation being readily brought on, and the weak parts forming the cicatrix giving way by ulceration, and forming again a very troublesome sore." 71.

The treatment, in these cases, is similar in kind, but more active in degree than in the others. The patient must be bled, vomited, purged, kept in the recumbent position, and local cold applications used as long as they are agreeable. When that ceases to be the case, fomentations are to be resorted to, and abandoned as soon as suppuration is established. Deep-seated inflammation requires leeches, but in much greater number than people are in the habit of applying in this country. The roller and graduated compress are the best means of cure in the subsequent stages, with change of air, and friction to the whole limb extremely actively employed.

When there is but one opening to a gun-shot wound, it is incumbent on the surgeon to ascertain, if possible, where the ball or any foreign bodies may have lodged. This is not quite so necessary where there are two openings. It is not uncommon for pieces of shell or grape shot to lodge wholly unknown to the patient, and are only discovered at a subsequent period, when much time has been lost and misery endured.

The finger, which is the best probe, should be gently introduced in the course of the ball, to its utmost extent, the hand of the surgeon being, at the same time, carefully pressed upon the opposite side where the ball may be expected to lie. The limb during this examination should be thrown, as nearly as possible, into the position which it occupied at the receipt of the injury. Where the finger cannot reach, a metallic or firm elastic bougie, or long silver probe, may be used.

All surgeons know what discussion there has been respecting the extraction of balls. The older surgeons, though anxious to remove these missiles, did not resort to extensive or deep incisions for that purpose, depending more on dilating and extracting instruments—enlarging the wounds only when they could not effect their object without it. These forcible dilatations increased, of course, the sufferings of the patient, and rendered more severe the subsequent symptoms. Paré in France, and Wiseman in England, made incisions—the former large and deep ones. This practice was advocated in France, and opposed in England, about the year 1792, by Baron

Percy and John Hunter. The experience of the Peninsula proves that John Hunter's opinions were correct, as far as they relate to wounds in muscular parts; but they led to a dangerous error—"that of neglecting dilatation under circumstances in which it was absolutely necessary." The practice, during the Peninsular war was, never to dilate without a precise object in view, which might render an additional opening necessary. "This opening must always, consistently with this principle, have been carried through the facia of a limb, and was truly a dilatation, whilst those incisions which were formerly made through the skin, or indentations in the edges of the facia, were entirely abandoned." The necessity for dilatation, in short, must first be seen, when the operation follows of course.

"I know not a stronger instance exemplifying this, than that of a wounded artery, which all admit to be a fair case for dilatation. Suppose, then, that a man be brought for assistance with a wound through the thigh, in the immediate vicinity of the femoral artery, and which, he says, bled considerably at the moment of injury, but which had ceased; is the surgeon warranted in cutting down upon the artery and putting ligatures upon it on suspicion? I believe every man in his senses will answer, no; the surgeon ought to take the precaution of applying a tourniquet loosely on the limb, and of placing the man in a situation where he can receive constant attention in case of need; but he is not authorized to proceed to any operation unless another bleeding demonstrates the injury, and the necessity for relieving it. By the same reasoning, incisions are not to be made into the thigh on the speculation that they may be hereafter required." 53.

Mr. G. makes some acute remarks on Mr. John Bell's case of wounded arm, where the fascia was four times divided with great relief, but where the contraction of the arm and the spasmodic disease of the whole system always recurred as the fascia healed. Our readers know that by a random stroke of the knife the fascia was fairly cut across at the place where it is braced down by its connexion with the long tendon of the biceps muscle, and then only the patient was entirely released. Mr. Guthrie contends that this incision was only proper when the symptoms required it—and that it would have been bad surgery to have done it early, and by way of *preventing* the consequences which, in this instance, took place; because, says he, these consequences will *not generally* happen, and we must go by the general rule rather than the exception. Here our author very justly observes that—"the maxim which obtains with many surgeons that the effects will subside, if the original, or exciting cause, be removed, is erroneous;" and he aptly illustrates this error by the treatment in strangulated hernia in a state of high inflammation. Here we take off the stricture first by the knife; but if the surgeon, as is sometimes the case, thinks he has done enough by ordering a cathartic or an enema, he fails as a scientific practitioner. The part is in a state of high inflammation, and the removal of the stricture upon it only takes off one cause of increasing inflammation, but does little towards removing that which already exists.

"In an early stage nature may be able to act for and relieve herself after the obstruction has been removed; but in a later one, when the inflammation is fairly established and running on towards gangrene, it is in the same state as any other common inflammation, and requires the same energetic mode of cure. When, then, I read, and have heard it said, that bleeding is of little or no use in strangulated hernia, of recent origin in young robust persons, or even in others, neither before nor after the operation, I am obliged to confess with regret, that surgery is never more degraded, than when it appears to be adorned with greatest splendour; that in no instance does surgery become so entirely an art as in this, where it especially deserves to be considered as a science. I have at this moment a preparation before me, in which the operation was successfully performed, but the patient was lost for want of medical treatment; and I firmly believe that the greater number of cases which are lost without mortification taking place, are lost from the same cause; but this is not peculiar to the operation for hernia. It was very common a few years ago, after all the great operations of amputation, and in many others of equally dangerous tendency." 88.

When inflammation, then, takes place in a wounded limb, general and local bleeding is to be used, cold or hot fomentations applied, purgatives are to be administered, with nauseating doses of antimony. If, on introducing the finger, the parts seem tense, and if the inflammation, pain, and fever run high, "an incision is to be made by introducing the knife into the wound, and cutting for the space of two, three, or four inches, according to circumstances, in the course of the muscles, and carefully avoiding any parts of importance." The same should be done at the exit opening, if mischief be impending; not so much for the purpose of loosening the fascia, as to abstract blood locally, and make a free opening for the evacuation of effused fluids. This our experienced author has done frequently with the most marked success.

In illustration of this principle we shall here introduce an interesting case from the *second edition* of Dr. Hennen's excellent work.

"A sergeant of dragoons was shot through the external part of the thigh at Waterloo, and was dressed for the first week by a Belgic surgeon. The lips of each orifice, which were plugged up with charpie, had been scarified in a radiated manner to about half an inch deep, as he said; but were nearly healed on my seeing him. Shortly after, heat, pain, and tumefaction took place in the limb, attended with considerable fever and great derangement of the head and stomach. This at last proceeded to such a degree, that the assistants requested me again to examine him, which I did on the 14th day. I found one orifice still open, and that some superficial scarifications had been repeated, and the limb fomented, but without effect; it was extremely tense, hot, and painful to the touch; it could not be moved without great uneasiness; the lower part of the limb, from the knee down, was œdematous, while the thigh itself was swollen up to the external trochanter; interiorly it was less so, but rather puffy. I made a long and deep incision from the trochanter nearly down to the knee, completely through the fascia, and about the centre of the limb I dipped almost to the bone. So far from this occasioning pain, the man begged me to go on; and, although there was but a very slight discharge of matter from the wound, he felt easier within an hour. The bleeding from the part was encouraged by warm fomentations; and in five days the sergeant was able to walk about, and was soon after discharged convalescent." *Hennen's Military Surgery, p.* 70.

The knife, Dr. Hennen remarks, is often indispensable in our search after balls, splinters, and bleeding vessels. "It is also useful in relieving the strictured state of the parts tied down by fasciæ, when that stricture is forming or formed;" but neither does the stricture form in all cases, nor does early scarification prevent it, because the tumefaction depending upon violence of injury, locality, and circumstances of constitution and treatment, the scarified parts may, and often do, heal before the occurrence of the contingency for which it has been employed.*

Our continental brethren appear still to adhere to the principle of scarifying wounds, by way of preventing inflammation, especially when they are situated in tendinous and ligamentous structure, as the following extract from Messieurs Laurent and Percy will shew.

" Toutes les parties du corps recouvertes d'une aponévrose dense et serrée, telles que celles qui se trouvent à la région postérieure du cou et de la colonne rachidienne, l'épaule, l'avant bras, la paume de la main, la partie supérieure externe de la cuisse, la jambe et la plante du pied doivent être incisées. Le chirurgien se servira à cet effet d'un bistouri boutonné, qu'il conduira sur son doigt indicateur préalablement introduit dans le trajet de la balle, ou sur une sonde cannelée, si celui-ci se trouvait trop étroit pour admettre un doigt. Il incisera de dedans en dehors tout le trajet de la balle, et il coupera l'aponévrose dans une étendue beaucoup plus considérable que les parties soujaçentes, en se conformant au précepte donné par Ambroise Paré de la scarifier dans tous les sens, afin de prevenir la hernie des muscles; l'anatomie guidera son doigt dans ce travail en sous œuvre, et l'empêchera de léser des vaisseaux et des nerfs qu'il est si important de respecter. Une partie qui ne serait pas recouverte d'aponévrose, mais dans laquelle on soupçonnerait qu'un corps étranger, qu'on ne pourrait extraire sur le champ, parce qu'il aurait perdu ses rapports avec l'ouverture extérieure, serait également incisée, afin de laisser un libre cours à la suppuration qui finit le plus souvent par

---

* Dr. Hennen observes in a note that these observations are confirmed by the experience of the venerable Dr. Jackson, who published similar opinions so long ago as the year 1790.

dégager la balle, et la ramène à la portée des instrumens destinés à en opérer l'extraction." *Dict. des Sciences Med. tom.* 43, *p.* 64.

Mr. Guthrie observes that the practice of dilatation is often highly serviceable in the subsequent stages of gun-shot wounds where sinuses form, and are tardy in healing. A free incision is also useful when parts are unhealthy, although there may not be any considerable sinus. The necessity of free incisions, where the bones are broken, need not be insisted on.

A punctured wound extending to a considerable depth labours under great disadvantages, and generally in proportion to the smallness of the instrument, and the difference of texture through which it passes.

"As a punctured wound cannot be changed into an incised one by incision, and the object of it, which would be union by the first intention, cannot be effected, an incision, in the first instance, may do harm, and will often be unnecessary; for many bayonet wounds through muscular parts heal with little trouble, and it is time enough to act when assistance seems to be required. Cold water should be used at first; care should be taken not to apply a roller or compress of any kind over the wound, and the matter should be frequently pressed out. When suppuration is established a roller should be applied above and below the wound, and an evaporating poultice upon it, if cold be found uncomfortable." 91.

Although some fortunate instances occur of balls lodging in bones without fracturing them, or producing much inconvenience, yet these exceptions are sadly counterbalanced by the results of other analogous instances.

"If a ball lodge in the head of a bone, and is not removed, it generally causes caries of the bone, disease of the joint, amputation, or death. If in the shaft of a long bone, necrosis for the most part follows, with months and years of misery. On a flat bone, caries is equally the result, and if it be surrounded by large muscles, sinuses form in various directions, contractions of the limb take place, and the patient drags on for years careless of life and ready to submit to any thing to obtain relief." 91.

Mr. Guthrie considers it a good general rule that balls should never be allowed to remain in bones. But there is often great difficulty in extracting them. Here Mr. Guthrie relates the fortunate case of Lieutenant Colonel Dumaresq, who was wounded in the battle of Waterloo by a musket ball, which passed through the scapula, penetrated and was lost in the chest. The thoracic inflammation was almost ungovernable, and he barely escaped with his life. A swelling was then discovered in the axilla, and was ascertained to be the ball, lodged in the rib, and is still there with a quantity of bone matter around it, while the Colonel enjoys good health. Where balls are discovered on the opposite sides of limbs, and at an inch or so distant from the surface, it has been recommended to allow them to remain, rather than to extract them immediately. But Mr. Guthrie has cut out great numbers of them, under these circumstances, and never found any inconvenience ensue :—on the contrary, the minds of the patients were relieved, and an opening was obtained for the evacuation of any matter formed in the long track of the ball. On the other hand, we are not to cut two, three, or four inches into the opposite side of a limb, for a ball which cannot be distinctly felt through the integuments or soft parts.

Mr. Guthrie here draws the attention of the surgical profession to a rare and suddenly fatal termination of gun-shot wounds, of which he has only seen a few cases. We shall introduce one as a specimen.

"On the retreat of the army from Fuente Guinaldo in 1812, a smart affair took place at the convent of Saca Farte, between the advance of the French, and the cavalry and the fourth division of the British army under Sir L. Cole, to which I belonged. The wounded accompanied me to Sabugal, on the heights near which we offered battle. Among them was a man, a stout handsome soldier, who had been shot through the right thigh, the ball entering below the femoral artery, passing through and outwards close to the bone; this wound went on remarkably well for near a fortnight, so much so that the man had actually got up and walked about. I saw him at one o'clock, and, as he was standing, desired him to keep himself quiet; he answered, he felt quite well. In the evening, Mr. Mahoney, now surgeon of the Fusiliers, who occupied the same quarters with me, reported that the man was suffering some pain, and that he had ordered him an opiate and a poultice. He died early in the morning, having complained a good deal in the night, but not sufficiently to induce the orderlies to call Mr. Mahoney, until symptoms of approaching death alarmed them. I examined the limb carefully within twenty-four hours of the man's being in comparative health. On the day previous to his death, the wound looked favourably, there was little or no inflammation, the limb was soft, and he was capable of walking, and conceived himself comparatively well. Inflammation came on in the night, internally, deep, and hardly affecting the skin with redness: on dissection the thigh appeared swelled, although not particularly so, but on cutting deeply through the fascia in the course of the wound, the whole thigh seemed so stuffed, or gorged with blood, that the texture of the parts, muscular as well as cellular, was soft, and readily giving way to a moderate pressure of the fingers; I can only compare it to the appearance of a part just falling into a state of gangrene." 99.

To check so rapid and dangerous an affection as this, Mr. Guthrie thinks that leeches should be applied in great numbers, after an incision has been made into the part. General depletion is then to be resorted to, "and one effectual bleeding, so as to cause syncope, will be most serviceable."

Hitherto Mr. Guthrie has only considered the treatment as applicable to healthy inflammation, in the treatment of which we act decidedly to command, as it were, success. In bad habits we are forced to act as circumstances may require. The phlegmonous and erysipelatous inflammations may be considered as two extremes of a scale, admitting between them many modifications—the erysipelas itself being greatly modified by habit of body, season of the year, climate, and prevailing epidemic diseases. It is therefore impossible to lay down any fixed rule of practice in this disease. When the constitution is good, and the fever inflammatory, Mr. Guthrie uses cold applications with advantage—especially the liquor ammoniæ acetatis, diluted with water, or with the addition of a little spirit. But when the constitution of the patient is bad, or the fever of a typhoid nature, Mr. G. has found warm fomentations of poppy heads, &c. with a small quantity of spirit, the best and most comfortable application. Erysipelatous inflammation is by no means common in gun-shot wounds, because these injuries are generally inflicted on healthy subjects. But that species of the disease termed *erysipelas phlegmonodes*, in which the inflammation extends to the cellular membrane, forming abscesses with little adhesive inflammation around them, so that the fluid secreted is diffused through the part, " giving to the finger the sensation of a partially circumscribed swelling"—such kind of erysipelatous inflammation is certainly of very frequent occurrence—indeed is present more or less in every case of gun-shot wound, not ending in mortification, and where the inflammation extends deeper than the skin. Mr. Guthrie has no experience of the free incisions formerly recommended by Mr. Copland Hutchison, while surgeon of the Royal Hospital at Deal. We can confirm the truth and justice of Mr. Hutchison's remarks on this point by personal experience. We fully coincide with Mr. Guthrie in the following sentiment:—

" In all cases of gun-shot wounds, in which erysipelas supervenes, the treatment is to be regulated by the symptoms, and I believe the same principles ought to be applied to it in every circumstance or situation of life in which it may be met with." 109.

Mr. Guthrie here notices a most dangerous and insidious inflammation, sometimes, though very rarely, (perhaps not more than two or three cases in an hospital of a thousand men) accompanying gun-shot wounds. It occurs after the 10th day, and in the five or six cases which came under our author's notice, the wounds were in the upper extremities. The wound, after the 10th day, swells and becomes painful—the redness is of a pale colour, more resembling phlegmonous than erysipelatous inflammation, whilst the skin has a shining glossy appearance, the tumefied parts being slightly œdematous to the finger, which, to a certain extent, is resisted by the firmness of the parts below. The pain is not greater than in other inflammations, and is rather burning than throbbing. The constitution sympathizes, at first, only in a moderate degree.—the swelling and shining appearance extend up to the axilla. The patient can sit up, or even walk, and neither countenance nor pulse indicates the near approach of dissolution, which, in five cases, took place a few hours after the last visit, when the appearances were as above described. All that could be learnt was, that they got worse in the night—that is, the pain increased, with difficulty of breathing, ending quickly in death. The three first cases were not examined. In the fourth, Mr. Guthrie could discover nothing beyond inflammation of the veins, especially those leading to the axilla, the axillary vein itself being inflamed. To this the death was attributed at the time, as it is not an unusual occurrence in fatal cases after amputation. In the fifth case, the man died of effusion into the chest, on the injured side. In this the veins were not affected, as in the other case. In the sixth case, the danger was immediately apprehended.

" The man was bled, purged, vomited, and diaphoretic remedies were administered, composed of calomel, antimony, and principally opium. Poultices were applied to the wound, and cold applications to the remainder of the limb. The arm swelled nearly up to the axilla, and I fully expected it would have taken the same course as the others, but it did not do so ; the inflammation gradually subsided, the arm diminished, and re-assumed its usual appearance ; the exact time I cannot mention, having lost the particulars of the case. During this process the man's health declined, he suffered an attack of fever, became afterwards jaundiced, and died under symptoms of diseased liver. There was nothing wrong at his death about the arm which had been inflamed." 111.

Mr. Guthrie hazards a conjecture that effusion in the chest was the cause of death in all the five cases. As it is only conjecture there can be no harm in our offering one too. We think it highly probable that there was inflammation of the internal tunics of the arteries in the majority of the cases. The state of these vessels is not mentioned in either of the two dissections.

The last result of inflammation in gun-shot wounds which Mr. G. takes notice of, is mortification. There is some confusion of ideas among authors respecting mortification, sphacelus, and gangrene. Our author seems to make the chief distinctions bear on constitutional and local states of the disease. It may take place from excess of inflammation, arising spontaneously, or from external violence. Gangrenous inflammation always shews a weakness of part, whilst it may not demonstrate any of the constitution ;—" in which case the gangrene ceases with the destruction of the weakened part, and the separation between the dead and the living is duly effected." The efforts of Nature, when gangrene takes place from excess of inflammation in sound constitutions, are too strong for the powers of the part weakened by disease.

" We accordingly endeavour to moderate them in the first instance by venesection, cathartics, and diaphoretics, and to allay irritation in the part by the appropriate remedies. When mortification has, however, taken place, we find that the constitution becomes immediately sensible of it, not by means of the absorbents taking up the gangrenous particles, as Baron Larrey supposes, but through the nervous and sanguiferous systems, which have been principally implicated in the disease, and have been entirely the instruments by which nature has attempted to effect its suppression. The pulse sinks, the countenance becomes anxious, the shock which has been sustained is perceptible, and the surgeon now endeavours to revive the drooping efforts of nature, which has been vanquished in the struggle. If the powers of the constitution have been fundamentally good, nature is able once more to rally, and although the part is completely irrecoverable, still she is capable of surrounding it, as it were, with a line of circumvallation, preventing the further encroachments of the disease, until, by renovated efforts of a slower but surer nature, she is able to cast it off altogether. But if the powers of the constitution have been bad, it is precisely the reverse, nature seems to have completely exhausted her strength in the struggle and to be totally unequal to further resistance." 115.

The pulse sinks, pain ceases, the skin becomes cold, perspirations break out, the countenance is inexpressibly anxious, and death soon closes the scene. Amputation of a member, in such circumstances, is forbidden, and our author thinks wisely so, by all modern surgeons, since the operation of amputation is an injury which the powers of the constitution are incapable of bearing. Inflammation in the stump scarcely commences before it ceases in the death of the part.

"If the operation should have been performed on a person of better constitution and powers, the stump may go on well for a day or two, and then actual gangrenous inflammation supervenes, as in persons of deficient powers, which will be followed by death. The axiom in surgery is then a good one, 'that whenever the constitution of the patient is implicated, whenever the powers of nature are considerably exhausted, the operation of amputation should not be resorted to until the line of separation is fairly established.' Amputation in this state is performed only when a part of a limb is completely destroyed, it is done to shorten and relieve the operations of nature, and to render the remaining portion of it more serviceable, or useful to the patient. It is consonant to reason that this injury, which is intended to be a beneficial one in its result, should only be committed, when nature has so far recovered from the preceding struggle, as to be able to set up and support the new actions required of her; and the appearance of the line of separation is a fair proof of her capability to do so." 117.

Mr. Guthrie takes up pretty extensively the subject of traumatic gangrene. Most of our surgical readers are aware that Baron Larrey advocates the practice of amputation in these cases, before the line of separation is formed—that is, as soon as the death of the part, and the consequent loss of the limb is demonstrated. "The supervention of gangrene on the stump," says the Baron, "is not to be feared as in spontaneous gangrene, in which the line of separation is not established, because traumatic gangrene is only propagated by absorption, and an affection of the different textures, which swell in consequence of the continuity of vessels along which the disease successively passes. In fact, amputation done at the proper place arrests its progress and prevents a fatal termination of the disease. The efforts of nature are to be seconded by bark, wine, tonics, &c." The Baron supports his opinions by several successful cases of amputation, in which the gangrene was spreading when the operation was performed. Mr. Guthrie shews clearly enough that the ingenious Baron's *theory* is quite defective, and the results of his practice he accounts for on very different principles. A cannon ball, Mr. Guthrie observes, in striking a limb, destroys the life of the part, more or less, according to the extent of the injury." Thus if a blow be received on the middle of the leg, the bone be broken, the arteries divided, or rendered incapable of carrying on the circulation, mortification takes place in the foot, because it is deprived of its usual support.

"It is possible, however, that this may not follow immediately, as it may not be entirely deprived of blood, which passes into it in small quantity from the parts above, connecting it with the rest of the extremity. The parts immediately above those actually struck by the ball have received a very considerable shock, and their sensibility is much impaired; and, when any action takes place in them, they will sometimes be found unequal to sustain it, and, as the action attempted to be set up is inflammation, the failure of support causes it to fall into gangrene. It is, however, a failure of support not from want of power in the constitution, exhausted by a serious struggle, but from incapability of the parts to maintain it. The extent to which this debility of parts may extend is uncertain, and the limits to the mortification must be so likewise, if left entirely to nature, it is evidently a struggle on the part of the constitution to reanimate the drooping powers of the part, which are unable to bear the assistance attempted to be afforded them. Inflammation then precedes the mortification, the limb swells, and has every appearance, above the wound, as the disease advances, of humid gangrene. It began as a local disease, the part being simply unable to live, and nature, having received a shock, as I conceive, entirely through the nervous system, and not by the absorbents, endeavours by means of an additional supply of blood (as she invariably does in every case of injury,) to recover the parts in jeopardy, to renovate their strength. If the parts are capable of bearing this, healthy inflammation is established and the mortification ceases. The disease is, from the moment inflammation is *established*, no longer local, the constitution is beginning to be implicated; and, if the struggle be continued, it becomes a case of mortification, dependent, according to my principles, on constitutional causes. Nature seems to suffer in the deprivation of the principle of life whenever she becomes sensible of the death of any part of the body, and in a greater proportion than would seem to be commensurate with the supply of that part in a state of health; she becomes weakened, of course, more by the death of a part than by its amputation, and upon a principle connected with life, which we cannot explain. When the inflammation commences, the great point for observation is, whether the power of the part can or cannot maintain and carry it on to the healthy, adhesive, and ulcerative stages. If nature can accomplish this, she ought not to be interfered with; but if it appear that the part is incapable of supporting the efforts of nature, or, what is worse, that she is incapable of making them; is she to be allowed to exhaust herself in a fruitless struggle, or is assistance to be given, the instant the inability of the part is seen, and just as the powers of nature are displaying themselves? I have no hesitation in saying, that the disease is yet a local one, nature is only showing what she will do if properly seconded; and that, if her efforts are directed to sound parts, capable of sustaining them, she will be able to make a sufficient and successful struggle. Amputation, then, is to be performed in sound parts, to which the usual efforts of nature will be directed; and, if they be unbroken, or only impaired by the previous injury, the result will be fortunate; but the inflammation will not be sufficiently powerful to be able to stop at the adhesive stage, union will not take place to any extent in the stump; suppuration should therefore be encouraged as a natural consequence, and no more adhesive straps should be applied than may be sufficient to keep the parts together, so as to prevent retraction. Warm fomentations and poultices should be preferred to cold applications, and the ligatures should all be cut short." 124.

Dr. Hennen, in his very valuable work, makes many sensible remarks on this point. He shews that so far back as 1782, Mr. Curtis, a naval surgeon, advocated the practice, since brought into notice by Baron Larrey and others, of amputating in traumatic gangrene before the mortification stops. Mr. Curtis details one very sasisfactory and successful case where amputation was performed at the Madras Naval Hospital, while gangrene was rapidly spreading. Dr. Hennen, both before and after he had read Baron Larrey's book, repeatedly amputated, without waiting for the line of separation. "And although," says this candid and intelligent officer, "I was not uniformly successful, I have no reason to imagine that death was occasioned by a departure from the rule so generally laid down by authors." *Hennen, p.* 243, 2*d Edition.*

In respect to the long-entertained notion of the "wind of a shot" producing sometimes so much mischief, it is unnecessary to dilate. No well-informed surgeon now believes in any such thing. Where serious effects are produced, the ball has come in actual contact with the part, whatever marks it may leave on the surface. Baron Larrey's idea is not a bad one, viz. that if it be a spent shot (which is generally the case) and have a rotatory as well as a progressive motion, it may, as it were, "turn round the part, in the same manner as a wheel passes over a limb, instead of forcing a passage through it." We cannot follow our able and ingenious author through his acute remarks on various causes and states of gangrene, particularly from injuries of the large vessels and nerves, from cold, from pressure, &c. but must refer to the work itself.

"Having endeavoured to establish the principles on which mortification is supposed to depend, it will be unnecessary to enter in detail into the treatment. In all cases, except in excess of inflammation, the internal means should be to soothe and support the system. Bark I have not found useful, further than as a tonic, and given in such quantities as not to overload the stomach. Camphor, the carbonate of ammonia, opium, wine, brandy, I conceive to be better remedies. As topical applications, emollient and fermenting poultices are decidedly the best, alternating with mild spirituous fomentations. I have always seen scarifications do harm, when they approached or interfered with living parts, and stimulant applications are only admissible, on the common principles of surgery, as applicable to parts in a state of ulceration. Any thing, however, which keeps them clean, and tends to remove or destroy the fœtor from those which are dead, is beneficial." 148.

This section of Mr. Guthrie's work closes with official tabular and narrative reports, shewing the effects of a battle, and the relative mortality in different classes of wounds. In the first ten weeks after the battle of Toulouse, one-eighth of the whole wounded died, and one-eighth more might be considered as dying afterwards, or as unfit for service; "the permanent loss, after a battle, to the effective strength, varying from one-fourth to one-third of the whole number wounded. Of 1359 cases treated, including officers, only one artery required a ligature, the other cases of secondary hæmorrhage having occurred as complications of compound fracture, rendering amputation necessary."

The second section or division of our author's work is on gun-shot wounds accompanied by lesion of the larger nerves. It has fallen to the lot of our author to see a great many wounds of this description—many of them productive of the greatest sufferings for years together. Physiologists are all aware, that if a large nerve be injured, the pain or inconvenience is not felt at the place of injury, but in the part to which the ramifications of the nerve are distributed. If it be completely divided, total loss of sensation and motion is the consequence—if only injured, the effect is pain in the part supplied by the nerve—which part has a greater susceptibility for stimuli than natural, with diminished capability of bearing them, partial loss of sensation, and still more of motion. The part of the nerve actually injured offers little or no impediment to the healing of the wound, which is generally effected in the usual time. The consequences which follow may be local or general; and are frequently of the most distressing nature. When a great nerve is divided, the extremity loses motion and sensation; yet the circulation appears to go on uninterruptedly, the size of the vessels, however, somewhat decreasing. The limb shrinks, the animal heat is reduced, and the power of resisting changes of temperature is nearly annihilated. The circulation in the capillary vessels is easily obstructed—the process of inflammation is not carried on in the same way as in health—it more rapidly passes over the adhesive stage and runs on to the ulcerative; but is not prone to pass into the gangrenous state, as in paralysis from internal causes. The absorbent system also seems, like the sanguiferous, in some measure independent of the nerves going to the part, its functions being carried on pretty regularly.

"An officer received two balls at the battle of Salamanca; one passed through the knee-joint, the other through the upper part of the chest, near the shoulder, but underneath the clavicle, dividing, as was supposed, the nerves going to the left arm, which was immediately deprived of the powers of sensation and motion. Suppuration occurred in the knee-joint, inflammation had taken place in the chest, he had a very troublesome cough, hectic fever had supervened, and this gentleman seemed to be almost without hope, when my opinion was requested. It appeared to me, after careful examination, although the lung of the left side had been affected by continuity of inflammation, and was still suffering from its consequences, that the hectic fever had principally been caused by the disease of the knee-joint, the amputation of which offered the only chance of relief. This appeared to be, and was, a very strong measure; but no intermediate one could be resorted to with any hope of success; and, after having the true state of the case made known to him, the patient decided that he would have his leg removed above the knee. The operation was done, and succeeded. He slowly recovered, and the wounds healed, but the arm remained in the state I have described. Eight years have elapsed, and he is now grown stout, is in excellent health, but the arm remains nearly the same. He has within the last two years perceived some sensation in the course of the muscular or spiral nerve. He has also been indicted for a rape; but the magistrates, very properly, however they might admit the attempt, would not consent to the admission of the fact." 159.

Here Mr. Guthrie relates some cases illustrative of the foregoing principles, and refers to others, particularly to those published by Dr. Denmark and Dr. Hennen. The latter gentleman has made many interesting remarks on this class of lesions; some of which we shall here notice. Dr. Hennen considers the mechanical injuries of nerves as, for the most part, entirely beyond the power of art to relieve effectually.

"A very common and most distressing set of sensations are the shooting pains and sympathetic feelings, referred by the patient to the fingers or toes of an amputated limb, which in some persons exist for months, or even years, after the operation. In some, cold or damp weather, lightning, or an electric state of the atmosphere, or an easterly wind, will produce it; in others, mental agitation, violent bodily exertion, intense thought, or excesses, particularly in venery, are sure to bring it on. In some instances it can be traced to no obvious source, in others it very clearly depends upon mechanical irritation." 191.

The 33d case in Dr. Hennen's treatise, of a general officer, is very interesting; but as it was stated in the first edition of this meritorious officer's work, it is pretty generally known to the profession.

"Œdema," says Dr. Hennen, "is a very frequent consequence of gun-shot injuries of the extremities, and is generally complicated with pressure of the lymphatics, or injury to the nerves, either immediately, or from the tumefaction of the parts from inflammation. By the use of gentle friction, with moderately stimulant embrocations, succeeded by the local shower-bath, and the subsequent application of a firm flannel roller, this troublesome symptom will be in general benefited after some time. I have also derived essential relief from the distressing numbness of the fingers in such cases, by the frequent evaporation of sulphuric æther upon the part. I have never noticed injuries of the lymphatic vessels themselves, unconnected with general affections of the limb." 196.

When a nerve happens to be included in a ligature, the latter is long in coming away, and is frequently broken off close to the knot, leaving the noose behind, and causing great misery to the patient. In Dr. Hennen's case (that of Sir George Cooke) the first attempt at clearing the ligatures was attended with excruciating pain, not referred to the stump itself, but to the finger, thumb, wrist, elbow, or even external skin of the lost arm, as one or other ligatures might be handled.

"Once only," says Dr. Hennen, did I ever know him refer his pain to the seat of the sensorium itself. On that occasion, from using an artery forceps to the ligatures, on which the slide moved rather stiffly, I exerted a greater force than I had intended. He convulsively put his hand to his head, expressed a sense of exquisite pain in his brain, involuntary tears dropped from his eyes, a paralytic contraction momentarily affected his mouth, an universal paleness spread over the uncovered parts of his body; and, although unusually tolerant of pain, and of a most remarkable equanimity of temper, he uttered a piercing cry, and exclaimed, 'that the agony in his head and neck was insufferable.' The state of collapse was so great that I was obliged to send an aid-de-camp instantly for volatile alkali, and a glass of Madeira, by which he was soon relieved; but the painful sensation, and the prostration of his strength, continued through the day. A British Admiral was present on this and various other occasions, and observed to me, after I had confessed my inability to explain, even to my own satisfaction, the cause of all these sensations, "that he never saw the General dressed without applying mentally to the wonderful sympathy manifested on those occasions, the expression of Pope, 'it lives along the line.' I believe we must be content with the fact, without seeking for the explanation." *Hennen, p.* 192, *2d Edition.*

Full a year elapsed before the last ligature came away. The General's health did not suffer, and no incisions or violent means were used. To account for the difficulty of detaching ligatures from nerves, we must recollect that the changes which take place in the extremities of these last, in a stump, are the reverse of those which occur in the sanguiferous vessels or bone itself. In a nerve the cut extremity swells, assumes a bulbous and oval form, becomes firmer in its structure, and permanently increased in size. It is evident that a ligature on such a part as this can never be detached till it becomes decomposed. Dr. Hennen remarks that;—" an experiment of placing a ligature on the axillary plexus, or any single nerve in the dead subject, will shew what an obstinate resistance is offered, *after the protrusion of the medullary substance*, by the subsequent puckering of the tough investing membrane which, during life, will not admit of the ligature sliding off, either until the part is absorbed in course of time, or the material of which the ligature is composed undergo some decomposion." 194.

Nerves, when wounded, often cause very distressing sensations, which cannot always be traced to the immediate seat of injury, in consequence of the local and general derangements produced by the wonderful sympathy and communication between all parts of the nervous system.*

Here Mr. Guthrie relates the case of a cavalry officer who received a wound from a musket ball in the back part of the right thigh, near the trochanter major, which lodged, but apparently without injuring the great sciatic nerve. The wound healed, but the limb remained weak, and liable, occasionally, to intolerable pain, yet without any privation of sense or motion. This pain was felt principally in the sole of the foot, and outside of the leg, in the direction of the fibular nerve. Cramps, as the patient terms them, are frequently attacking him, by day and by night, especially on any change of weather, or any irregularity in living. If in bed, he is obliged to jump out, and put his foot on a cold stone, which affords him momentary relief.

We cannot follow Mr. Guthrie through the remaining observations on neuralgic affections arising from injuries or spontaneously, especially as most of them are taken from works well diffused among the profession. We must also pass over, at least for the present, a great part of Mr. Guthrie's work now reprinted and improved, in order to give some account of a particular section dedicated to the subject of amputation at the hip-joint. Before entering on this subject, however, we shall glance cursorily at one or two points in surgery, which we have neglected to notice in their places.

At page 247 *et seq.* Mr. Guthrie discusses a very interesting point in surgical pathology—namely, those determinations to, or irritations in, particular viscera after operations.

---

" * Stumps are frequently subject to severe spasmodic affections, which extend towards the trunk in the course of the nerves, and are often productive of great alarm. I know one gentleman who suffers very frequently from them, and as I conceive from two causes, exposure to cold, or derangement of the digestive organs; and he obtains relief from the use of purgatives, and the application of leeches and warm fomentations to the part, followed by the use of stimulants and narcotics." 170.

"When the inflammation attacks the lungs, the approaches of it are very insidious, the soldier does not suffer sufficient to make him apply for particular assistance, as he labours under fever; and when the disease has advanced to that point that the attention is especially drawn to it, the time for assistance is past, and the disease shortly proves fatal; in some instances apparently by suffocation. The lungs on dissection are found full of blood, and firmer than usual, occasionally pus is formed in them, or there is effusion into the air-cells, and into the cavity of the chest." 248.

Mr. Guthrie considers that these accidents are much more prone to occur after secondary than primary amputations—another reason for preferring the latter. Our author complains that although these observations were published in 1815, Mr. Charles Bell, in 1817, states that the point in question is "one of the many subjects authors have neglected to treat of." We are sure Mr. Bell would not wilfully withhold any priority of claim that might be fairly due to a cotemporary practitioner. We are the more inclined to this belief as we observe that this eminent teacher has ceased to deliver certain doctrines respecting the great difficulty of compressing arteries, so as to effectually restrain the flow of blood from them, long maintained by his illustrious brother, himself, and we believe most other surgeons in civil life. It is unquestionably by our army and navy practitioners that the following erroneous position of Mr. John Bell has been refuted.

"He says, page 415 of his Principles of Surgery, 'I will repeat with confidence what I have frequently affirmed, that it is one thing to suppress the pulse in the lower part of the limb, and another thing to stop the pulse in the great artery. I have tried in great operations, near the trunk of the body, to stop the blood by pressure; but though I could suppress the pulse of the femoral artery with my fore-finger, I could not command its blood with the whole strength of my body."

Contrary to this, we can confirm, by numerous observations, the following statement of Mr. Guthrie:—

"I have no hesitation in declaring, and I am supported in the assertion by all the surgeons of extensive practice in the British army, that when the pulse is suppressed in a great artery, the flow of blood is completely restrained for every purpose in surgery. I will even say, that the flow of blood may be entirely suppressed, and yet the pressure upon the subclavian artery above the clavicle, shall be so moderate, that the instrument will not leave a mark upon the skin discoverable after twenty-four hours. I do not assert this without solid foundation, for I have seen the inguinal and subclavian arteries compressed and divided very many times, and I have had the femoral and axillary arteries as often between my fingers; but, I never saw blood projected one inch from the orifice of these vessels without a pulsatory motion being evident." 343.

Mr. Guthrie does not doubt the correctness of Mr. Bell's ments, when he mentions his inability to suppress the circulation in the *cases of aneurism.* He means to assert only "that the passage of blood through a healthy artery can be effectually prevented by moderate pressure; and that when the pulse has ceased in a large artery, the surgeon may divest himself of all fear of hæmorrhage." This fact is now familiarly known throughout our army and navy medical brethren.

It was our intention to have noticed the important subject of *excision of the head of the humerus,* so ably discussed in Mr. Guthrie's work; but as the observations of Mr. Bell and those of Mr. Guthrie are now fairly before the public, we leave that public to decide between these able surgeons. We have not space to state the question in a full and proper manner, and without such a step—

Non nostrum inter illos tantas componere lites.

*Amputation at the Hip-Joint.* This formidable operation has been seldom performed, surgeons having agreed to apply to it the epithets dreadful and horrible, and consider it as hopeless—nay, as an unnecessary cruelty. "Fear," says our author, "has so much pervaded the minds of all (surgeons in civil life) that it is generally regarded in domestic surgery with as much horror, as the attempt at putting a ligature on the external iliac artery would have been a century ago, if such an operation had been proposed." The opinions of military surgeons, supported by stubborn facts, are dispersing these prejudices; and few surgeons of any pretentions to eminence would now hesitate to perform the operation in question, under proper circumstances. Pott *saw* it performed in London, but appears to have formed a resolution never to perform it himself—unless on a dead body. Without doubt it is a formidable operation; but, when there is no other chance for life, we must apply the precept of Celsus:—"Nihil interest an satis tutum sit præsidium, quod *unicum* est." It appears that M. La Croix, in the year 1748, completed an operation of this kind, which Nature had nearly effected. A boy, thirteen or fourteen years of age, was brought into the Hotel Dieu with gangrene spreading up both thighs. A line of separation, however, formed around the right hip-joint, and the limb was nearly detached by the efforts of Nature. M. Le Croix completed the detachment with a pair of scissors, and the boy was so well on the fourth day, that the surgeon proposed to amputate the other limb, there being sufficient space left for that purpose. This was performed, and the boy was doing well, but being seized with fever, he died fifteen days after the operation.

Dr. Kerr of Northampton published a case of this kind in 1779. A girl had a large abscess near the great trochanter, with disease of the joint, and hectic fever. He amputated at the joint, but found the acetabulum carious, as well as a portion of the os innominatum. She died hectic on the 18th day after the operation.

Baron Larrey has performed amputation at the hip-joint seven times. In the first case there was every appearance of a favourable result, but a rapid march of twenty-four hours' duration marred their hopes, and probably caused the death of the patient. The second case was Mons. Bonhomme, wounded by a splinter of a shell at the seige of St. Jean D'Acre.

"The muscles were either torn or carried away from a great part of the circumference of the thigh, the femoral artery was torn about five or six fingers' breadth below the crural arch, and the femur was broken as high as the great trochanter. He had lost a considerable quantity of blood, and was very much weakened. I even thought he would die in a few minutes, if the removal of the thigh was not immediately accomplished. He passed the day and night after the operation in as quiet a state as could be desired. I gave him some antispasmodic draughts, some cooling drinks, and weak broth with a little wine. The next morning the dressings were wet with a reddish-coloured serous discharge, without swelling, pain, or tension in the stump. He was easy during the night, and slept well for three hours. The third day the dressings were removed, and he passed the day very well; the bowels were regular, and he had a desire to eat. I gave him some rice gruel (pottage) night and morning. On the night of the third to the fourth day, he had some slight febrile symptoms accompanied by throbbing in the stump, and general heat of the body, which was succeeded by a plentiful perspiration, ease, and sleep. In the morning I found the dressings soaked with a purulent discharge. The flaps had already united the half of their extent, leaving at their upper and under extremities an opening about two inches long, where I had brought out the ligatures. On the fifth day every thing appeared to be going on as well as possible. The matter discharged from the upper and lower openings was of good quality and quantity. On the sixth day his situation was still very favourable, and I had every reason to expect a cure; but the crowded state of our hospital, and the impossibility of separating even the most severe cases of wounded from the other sick, were the cause of an unfortunate occurrence the next night, which the particular nature of our situation did not enable me to guard against." 305.

This accident was the introduction of plague into the hospital, of which the unfortunate officer died.

The 3d case was a drummer who had his thigh carried away by a splinter of a shell, and who was operated on immediately by the Baron. The return of the army to Egypt took place, and this young lad died on the journey. The Baron performed it twice unsuccessfully after the battle of Wagram. In the Russian campaign he also performed it twice. In the first of these the patient lived till the 29th day, when he was carried off by dysentery.

The second took place after the battle of Mojaisk, and the man recovered, but never having reached France, there is some doubt on the subject. M. Baffos, a Parisian hospital surgeon, performed this operation on a boy of seven years of age, on the 3d January, 1812, in the presence of several surgeons.

"'M. Danyau, standing on the left side of the patient, compressed the artery with the thumb of the right hand. Holding the thigh with my left hand, I moved it gently backwards and forwards to enable me to observe the spot nearest to the joint where the knife ought to enter; which having ascertained, I plunged a sharp-pointed straight knife, eight inches long, and six or seven lines broad, into the anterior and superior part of the thigh, external to the artery, and brought it out directly opposite behind. I then cut along the bone for the breadth of four fingers, when, turning my knife, I made a horizontal cut, by which I completed the internal flap. M. Danyau grasped this in his hand, and completely commanded the flow of blood. I now exchanged my knife for a straight bistoury, with which, at one stroke, I cut into the capsular ligament and divided the round ligament, luxating at the same time the head of the femur by a strong abduction of the thigh. Resuming my first knife, I carried it behind the head of the bone, and made a horizontal incision outwards, on a level with the top of the trochanter, which completed the external flap. I pulled out the artery with the forceps, and tied it with a double thread; and then secured the lesser arteries that bled in succession; but there were not more than seven or eight requiring the ligature.

"The operation was performed in thirty or forty seconds, not including the time required for securing the vessels; and the quantity of blood lost was supposed to be something less than a porringer full (palette.)" 310.

The little patient did well, and was about to be discharged, when on the 63d day, he was seized with fever and diarrhœa—the cicatrix opened out—and the boy died.

Mr. Guthrie thinks that the principal accident rendering necessary this operation, will be a fracture of the head or neck of the bone, with or without a wound of the great vessels, or some artery causing hæmorrhage and stuffing of the thigh with blood. Few surgeons, he observes, would think of performing this operation for a wound by a musket ball, though such cases may occur. A grape or small connon shot may strike the fore part of the thigh, and, without touching the inguinal artery itself, in its passage to the neck of the femur, wound some large arterial branches, causing considerable hæmorrhage. The wound may not be large, and yet the chance of saving the life of the patient may be small indeed. Mr. Guthrie recollects two cases of this kind, one after the battle of Vimeira, the other at Salamanca, by a large ball which shattered the neck of the femur and the body of the bone below.

"I did not see this person (an officer) for near forty-eight hours after the injury, but was informed that on his first presentation for assistance, an artery, supposed to be a large branch of the femoral, had thrown out its blood per saltum, and was stopped by pressing some lint on the wound. The limb soon swelled to nearly twice its natural size, with much external inflammation. The patient himself thought his case desperate, as did every one about him, and declared his willingness to submit to any thing that might be proposed; but the time for operating was past, even if any operation could have been agreed upon.

"After two months of severe suffering, in which there were even some prospects of life being preserved, this gentleman died." 314.

Our experienced author remarks that, when the femoral artery has been torn by a cannon shot, there is, at the moment, a great loss of blood, but the patient does not bleed to death; neither does he appear to die ultimately from the effects of the hæmorrhage. A very extensive injury of the soft parts of the thigh, if the bone be not broken, and the femoral artery not divided, does not authorize the operation, although the artery be laid bare for three or four inches of its course. This Mr. Guthrie exemplifies by an interesting case, of which we shall exhibit an outline.

Captain Flack was wounded in the trenches, at the siege of Ciudad Rodrigo, by a 24 pounder that struck the exterior and anterior part of the left thigh, carrying away the fore part of it from the groin to within a hand's breadth of the knee. The femoral artery lay bare at the bottom of the upper part of the wound, the sartorius and rectus muscles being carried away, and all the muscles on the outer and inner side of the thigh more or less mangled and torn. All the medical officers present declared he must die, unless the limb were removed. In compliance with this opinion, Mr. Guthrie proposed to tie the artery below Poupart's ligament, and endeavour to save flaps to cover the great trochanter, sawing off the bone below, as he has since done in several instances. If this was not found practicable, he intended to remove the limb at the joint. When the patient was placed on the panniers, however, he appeared to be dying, and the unfortunate officer was placed on a hay-mat in one corner of the hospital to quietly expire, a little lint being laid over the enormous surface of wound. Next morning, however, he was much recovered, and as his thigh became very painful, he was desired to keep it wet with warm water. In this state he remained till the day after the storming of Ciudad Rodrigo, when the advance of the enemy caused the removal of the wounded across the Agueda. Mr. Guthrie again found the suffering officer, and humanely had him conveyed to his own divisional hospital five leagues distant. Mr. G. daily expected to see the femoral artery give way, but nothing of the kind took place, though the slough from the whole surface of the wound soon separated, with great discharge of pus, the artery lying in a channel completely surrounded by it. Granulations soon began to shoot out, and by the end of three weeks the artery was covered in, although its pulsations were still visible at a distance. The sore gradually contracted, and he ultimately did well. This case is rather calculated to check absolute despair, than to inspire hopes in similar circumstances. The following unfortunate case will explain another state of disease in which the operation in question may be necessary.

"Private Mason, of the 23d regiment of infantry, or Welch Fusiliers, had his thigh amputated about its middle during the siege of Ciudad Rodrigo, and was sent to the divisional hospital at Aldea del Obispo, with the other wounded of the fourth division, those of the other part of the army being in general hospital. For some time he appeared to be doing well, when the wound became irritable, opened out, and sloughed on the under and inner part, with some hæmorrhage. Attention was paid to this both in dressing and searching for the vessel, which could not be found, for the stump ceased to bleed when opened and cleansed, and yet soon filled after the dressings were applied. Finding my endeavours to suppress the hæmorrhage fruitless, I determined on tying the femoral artery above where the profunda is usually given off, as it appeared to be a branch of that vessel that bled. This was effected, as I supposed, about two inches and a half below Poupart's ligament, with little disturbance to the contiguous parts. The hæmorrhage ceased from the stump, and I hoped all would do well. The next morning the bleeding suddenly returned, and about a pound of coagulated blood was removed from the stump. Pressure of the artery against the bone hardly commanded it, and the poor man earnestly begged something might be done to save him. The appearance of the stump had deteriorated very much in the last twenty-four hours, the ulceration was extending deeper between the muscles, and the prospect of the healing of the stump without much exfoliation of bone, even if the hæmorrhage could be suppressed, was but trifling. He willingly agreed to have the stump removed a little below the ligature on the artery, although he was aware his chance of surviving it was doubtful: but, finding himself much weakened from the loss of blood, he said to me, 'I must die, Sir, to-night, if I keep it, and I will take my chance.

"The artery being compressed against the os pubis, I carried my incisions to the bone, taking the ligature on the artery as the centre; and in doing this I observed a vessel equal in size to the one I had tied, running down a little distance behind and on the outside of it, which I immediately secured. The head of the bone was now removed from the acetabulum without difficulty. Fourteen vessels were tied; yet little blood was lost, as my two assistants, Mr. Cartan, now surgeon of the 8th regiment, and Mr. Loane, late surgeon of the 94th, the only professional men present, aided me in pressing on the mouths of the vessels with their fingers, until I could take them up in succession. The parts were brought together, and the integuments retained in contact by two sutures with the needle above and below, assisted by adhesive straps. The operation from first to last was completed in less than a quarter of an hour, and the man bore it heroically. I had even strong hopes of him for the first hour, but he gradually sunk, and died seven hours after the operation.

"On examining the original stump I found the femoral artery perfectly open where the ligature came off, but from this part it never bled. It was from underneath the blood came, and from some small branches of the profunda. This vessel was given off from the external iliac, or rather the external iliac divided into two equal branches, immediately after giving off the epigastric and circumflex arteries. There was the same peculiarity in the other limb, and the vessel acting as the profunda sunk into the thigh at the usual place. I have always thought this man would have lived if the amputation had been performed when the femoral artery was tied, which operation of course could not succeed, from the peculiarity in the origin of the profunda." 320.

The most common cases requiring the performance of this operation, at a late period, are, Mr. Guthrie thinks, compound fractures above the middle of the thigh, which have been badly or unsuccessfully treated, and in which the bone as well as the soft parts become a mass of disease, or cause such continued pain and irritation to the patient that the operation offers the only chance of life. The two cases that occurred in England, one of which proved completely successful, were of this nature. The unsuccessful case lived till the 30th day, and the account of it is drawn up by Dr. Emery, inspector of hospitals. We shall sketch an outline of it here.

Sebastian, a corporal in the Chasseurs Britanniques, received a wound in the left lower extremity, in Spain, in August, 1818; the ball entering the upper and outer part of the thigh, passing obliquely downwards and inwards, fracturing the os femoris, passing through it, and extracted about the centre of the sartorius muscle. In February he arrived in England, the wounds discharging copiously, the limb much indurated, and the patient in a low irritable state. The ossific deposition was so great that the thigh, both above and below the fracture, became considerably enlarged, the old original bone having grown carious to a great extent, attended with sinuses in the soft parts running up to the trochanter major, and downwards to the condyles. On the 18th July, 1814, a consultation was held on the patient, his body being much emaciated and reduced by hectic fever, night sweats, and diarrhœa, the extremities cold and œdematous, appetite gone, and the constitution evidently giving way. The patient consented two days after this, and the operation was performed in the following manner:—

"On the 21st, having agreed to the operation, the symptoms being more favourable, he was placed diagonally on a narrow table, supported by a strong man behind; the operation was then commenced by making an incision with a large scalpel through the integuments, beginning four fingers' breadth below the anterior superior spinous process of the ilium, which was carried with a convexity downwards on each side of the thigh, and meeting close to the tuberosity of the ischium: the adipose membrane was then separated from the fascia and drawn upwards; the femoral artery was next laid bare by dissection, and secured below the giving off of the profunda, by passing an eyed probe under it with a double ligature, which, being separated, were tied in two knots half an inch asunder, and the vessel divided between them; the vein was also tied, to prevent an effusion of blood from the limb. The scalpels were now laid aside, and an amputating knife applied close to the retracted edge of the skin, cutting obliquely down through the muscles of the thigh on the fore part and inside. In doing this the patient lost a few ounces of blood, though chiefly from the veins, which, with the arteries then cut, were immediately secured by ligatures. After this the incision round the thigh was finished, by dividing the remaining uncut muscles; which being accomplished, the femur was laid completely bare by dissecting them up with a curved scalpel, first on the outside, entirely above the trochanter major, and then on the inside, till the notch of the acetabulum was rendered perceptible, through which a double-edged bent bistoury was introduced into the capsular ligament, dividing with it also the round ligament, which was made to present itself by turning the limb outwards. The head of the bone was next extracted, the vessels taken up, and the operation finished by removing, with the same bent instrument, as much of the cartilage that lines the cotyloid cavity as could be got at, scarifying what could not be taken easily away, and detaching some ligamentous filaments and synovial apparatus. The wound was then cleansed, brought together in a straight line, and secured by four sutures passed through the cellular substance at equal distances, nearly close to the edges of the skin, supported by strips of sticking plaster, covered with pledgets of lint, common dressings, &c. and the whole made fast by a broad calico bandage, to which a cushion was attached, being fixed so as to press on the parts opposite, with a view of making them fill the cavity caused by the removal of the trochanter major, and act as a prop to the external and inferior side of the stump. He was immediately put to bed, and a draught composed of forty drops of tincture of opium and of spirits of nitre was given." 323.

The patient slept well the night after the operation, and during several periods of the succeeding thirty days he promised well, both as to constitution and wound. A bowel complaint, however, appears to have harrassed him the greater part of the time, and towards the close there were two or three hæmorrhages of trifling extent. On dissection, besides a highly morbid state of the parts engaged in the operation, the liver was found enlarged to the weight of seven pounds; of a straw colour externally, and like as though it had been parboiled in its internal structure. The stomach and jejunum bore marks of inflammation.

Mr. Brownrigg, surgeon to the forces, has thrice performed the hip-joint operation, in one case with complete success. The man received a gun-shot wound in the thigh, which fractured the bone close to the trochanter, in December 1811, near Meisda, in Spain. About a year afterwards the operation was performed, and the man is now living at Spalding, in Lincolnshire, perfectly well.

After the battle of Waterloo Mr. Guthrie performed this operation successfully on a French soldier, who had been left for several days on the field of battle, without surgical aid or sustenance. The detail is too long for us to attempt an abbreviation, and we therefore refer to the work itself for the particulars. The man is now in the Hotel des Invalides at Paris, and in very good health. The wound requiring the operation was by a musket ball, which entered behind, fractured the neck of the femur, and made its exit anteriorly, about four inches below the groin. In addition to his wounds, which had put on a sloughing appearance, he suffered from an extensive sore on the sacrum, which was caused by lying on the wet ground for five days.

As our limits are drawing to a close, we must be brief in our further *historical* notices of this important operation. Mr. Cole operated in this way, on the 6th April, 1814, after the unsuccessful attack on Bergen-op-Zoom. The patient was in a bad state of health, and only survived the operation 24 hours. Dr. Blicke's patient survived eight days. Mr. Brodie's and Mr. Carmichael's cases were also unsuccessful.

The cases alluded to prove, as Mr. Guthrie justly remarks, that the operation is not only necessary but practicable, "and that it may be effected with success under certain circumstances." If this be granted, which we apprehend it must, then the operation ought to be recommended "in every case in which it alone can bring relief, or offer a prospect of success." No man, consequently, should be allowed to die, without its being proposed to him; and if it be a case for primary operation, the sooner it is done, on the field of battle, consistent with propriety, the greater will be the chance of success.

We shall pass over Mr. Guthrie's observations on the difficulty of suppressing the current of blood in the larger arteries, since every military and naval surgeon has had repeated proofs how easily the inguinal and subclavian arteries, in a healthy state, can be completely controlled by even a very moderate degree of pressure. With the knowledge of this important fact before him, Mr. Guthrie objects to Baron Larrey's preliminary step, in the hip-joint operation, of tying the artery and vein, as being unnecessary and tedious. We shall give Mr. Guthrie's own method in his own words.

"The patient should be laid on a low table, or two field panniers placed together, covered with a folded blanket to prevent the edges giving pain, and properly supported in a horizontal position. An assistant, standing on the opposite side, and leaning over, should compress the artery against the brim of the pelvis, with a firm, hard compress of linen; such as is generally used before the tourniquet; he should also be able to do it with his thumb, behind the compress, if it be found insufficient. The surgeon standing on the inside, with a strong, pointed amputating knife of a middle size, makes his first incision through the skin, cellular membrane, and fascia, so as to mark out the flaps on each side, commencing about four fingers' breadth, and in a direct line below the anterior superior spinous process of the ilium in a well-sized man; and continuing it round in a slanting direction at an almost equal distance from the tuberosity of the ischium, nearly opposite to the place where the incision commenced. Bringing the knife to the outside of the thigh, he connects the point of the incision where he left off with the place of commencement, by a gently curved line, by which means the outer incision is not in extent more than one-third of the size of the internal one. The integuments having retracted, the glutæus maximus is to be cut from its insertion in the linea aspera, and the tendons of the glutæus medius and minimus from the top of the trochanter major. The surgeon now placing the edge of the knife on the line of the retracted muscles of the first incision, cuts steadily through the whole of the others, blood vessels, &c. on the inside of the thigh. The artery and vein, or two arteries and a vein, if the profunda is given off high up, are to be taken between the fingers and thumb of the left hand, until the surgeon can draw each vessel out with the tenaculum, and place a ligature upon it. Whilst this is doing, the assistants should press with their fingers on any small vessels that bleed. The surgeon then cuts through the small muscles running to be inserted between the trochanters, and those on the under part of the thigh, not yet divided; and with a large scalpel opens into the capsular ligament, the bone being strongly moved outwards, by which its round head puts the ligament on the stretch. Having extensively divided it on the fore part and inside, the ligamentum teres may now be readily cut through. The head of the bone is then easily dislocated, and two or three strokes of the knife separate any attachment the thigh may still have to the pelvis. The vessels are now carefully to be secured. The capsular ligament, and as much of the ligamentous edge of the acetabulum ought to be removed as can readily be taken away. The nerves, if long, are to be cut short, the wound well sponged with cold water, and the integuments brought together in a line from the spinous process of the ilium to the tuberosity of the ischium. Three sutures will in general be required, in addition to the straps of adhesive plaster, to keep the parts together; the ligatures are to be brought out in a direct line between the sutures, a little lint and some compresses are to be placed over the wound, and on the under flap, to keep it in contact with the cotyloid cavity, and assist the union of the parts. A piece of fine linen is to be laid over them, and the whole retained by a calico bandage put round the waist, and brought over the wound.

"It is recommended to pare the cartilage from the bone; and if this could be readily done, I would agree to it, but the cartilaginous surface of the acetabulum is not to be cut away without much difficulty and some time, which cannot be spared; for I consider the success of the operation to depend very much upon the quickness with which it is performed, not on account of hæmorrhage, but to avoid the shock the constitution receives from the continued exposure and irritation of so large a surface in the immediate vicinity of the trunk of the body. It is proved by experience to be unnecessary at the shoulder-joint; and will, I think, be found equally so at the hip." 354.

In conclusion, Mr. Guthrie states, that in February 1817, he amputated at the trochanter major, in presence of Mr. Astley Cooper, who compressed the artery at the groin. The limb was removed by a circular incision, nearly, however, in the manner above described. The bone was sawn through at the trochanter major, and the arteries tied afterwards, a ligature being placed separately on the femoral artery and on the profunda. The patient lost very little blood, and was not more than fifteen minutes under the operation. In this case matter formed in the stump in four different places, requiring four incisions of two or three inches in length, to give free vent to the discharge. At the end of six weeks he was walking about the streets on crutches. But being caught in the rain, in Bond Street, he was attacked with thoracic inflammation, of which he died.

Our limits are so far exceeded that we cannot notice the many improvements which the formerly published part of this work has experienced. Neither can we do justice to the new edition of Dr. Hennen's volume. These two works will necessarily be in the hands of all naval and military surgeons, as well as those surgeons in civil life who are at all in the way of operations. To these classes in particular we most urgently recommend the volumes before us, as highly illustrative of the exalted eminence to which BRITISH MILITARY SURGERY has attained, and exceedingly honourable to the officers who have effected such improvements in the surgical science of their country.

